# Aldosterone in Diabetic Kidney Disease: From Mineralocorticoid Receptor Antagonism to Aldosterone Synthase Inhibition

**DOI:** 10.3390/ijms27135664

**Published:** 2026-06-23

**Authors:** Juarez R. Braga, Joseph H. Holthoff, Luis A. Juncos, Ramakrishna Thotakura, Fatima Ayub

**Affiliations:** 1 Division of Nephrology, Central Arkansas Veterans Health System, Little Rock, AR 72205, USA; juarez.braga@va.com (J.R.B.); joseph.holthoff@va.gov (J.H.H.); ramakrishna.thotakura@va.gov (R.T.); 2 Division of Nephrology, University of Arkansas for Medical Sciences, Little Rock, AR 72205, USA; luis.juncos@freseniusmedicalcare.com; 3 FMC Global Medical Office, Medical Affairs, Fresenius Medical Care, Waltham, MA 02451, USA

**Keywords:** diabetic kidney disease, renin–angiotensin–aldosterone system, angiotensin-converting enzyme inhibitors, angiotensin receptor antagonists, mineralocorticoid receptor antagonists, aldosterone synthase inhibitors

## Abstract

Diabetic kidney disease (DKD) represents the single most common etiology of chronic kidney disease and end stage kidney disease globally, a burden that continues to expand in direct proportion to the worldwide growth of the diabetes epidemic. The pathogenesis of DKD is multifactorial, involving metabolic, hemodynamic, inflammatory, and fibrotic pathways. Among these, aldosterone has emerged as a key mediator of kidney injury, extending beyond its traditional role in sodium balance and blood pressure regulation. Through activation of both MR-dependent transcriptional processes and MR-independent signaling cascades, aldosterone drives a coordinated pattern of renal injury encompassing oxidative stress generation, endothelial dysfunction, podocyte damage, inflammatory cell recruitment, and progressive interstitial fibrosis. Current therapies targeting the renin–angiotensin–aldosterone system (RAAS), including angiotensin-converting enzyme inhibitors, angiotensin receptor blockers, and mineralocorticoid receptor antagonists, have significantly improved outcomes in DKD. Despite these advances, a considerable degree of residual cardiovascular and renal risk persists, attributable in part to the incomplete attenuation of aldosterone activity and the well-characterized phenomenon of aldosterone escape under sustained RAAS blockade. Aldosterone synthase inhibitors (ASIs) represent a mechanistically distinct therapeutic approach that targets aldosterone overproduction at its enzymatic source, potentially addressing both MR-dependent and independent pathways. Early clinical trials evaluating the efficacy of ASIs have demonstrated promising effects on blood pressure and albuminuria. This review summarizes the role of aldosterone in DKD pathogenesis, evaluates current therapeutic approaches, and discusses emerging evidence supporting ASIs as a potential addition to the evolving treatment landscape.

## 1. Introduction

Diabetic kidney disease (DKD) is the leading cause of chronic kidney disease (CKD) and end-stage kidney disease (ESRD) worldwide, representing a major and growing public health burden. The worldwide prevalence of diabetes mellitus is increasing at an accelerating rate, with projections exceeding 1.3 billion affected individuals by 2050, driven largely by increasing rates of obesity [1]. As a result, DKD is expected to become even more prevalent in clinical practice, posing significant challenges for nephrologists and healthcare systems. The pathogenesis of DKD is complicated and multifactorial, involving an interaction of metabolic, hemodynamic, inflammatory, and fibrotic pathways. As the understanding of the pathogenesis of DKD evolves, new therapeutic options are emerging. Despite advances in understanding these mechanisms, progressive kidney fibrosis remains the final common pathway leading to kidney failure. Among the various contributors, dysregulation of aldosterone has emerged as a particularly consequential contributor of DKD. Although ACE inhibitors and ARBs have meaningfully improved renal and cardiovascular outcomes through the suppression of RAAS activity, a substantial burden of residual risk persists in treated patients, reflecting the incomplete inhibition of aldosterone-mediated injury that conventional RAAS blockade achieves. In this context, increasing attention has been directed towards targeting aldosterone more effectively, including through mineralocorticoid receptor antagonists (MRAs) and more recently aldosterone synthase inhibitors (ASIs). In this review, we examine the role of aldosterone in the pathogenesis of DKD, highlight current therapeutic strategies, and discuss the evolving landscape of therapies targeting aldosterone synthesis.

## 2. Synthesis and Secretion of Aldosterone

**Figure 1:**
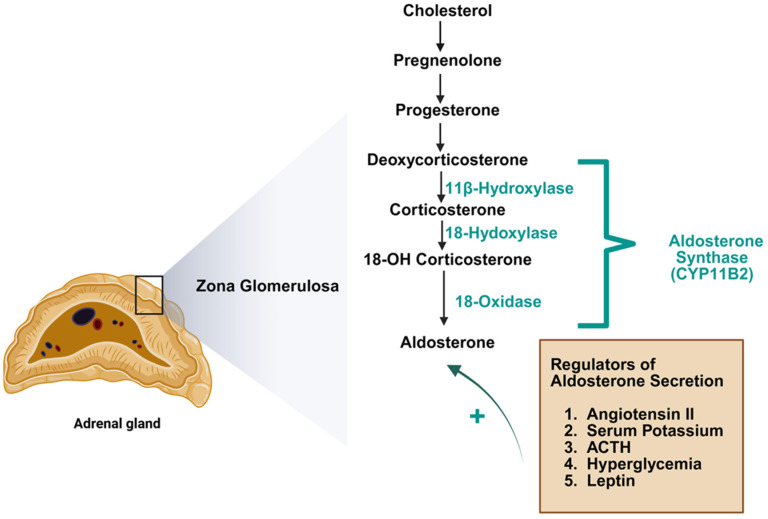
Aldosterone synthesis and regulation.

Aldosterone is a steroid hormone derived from cholesterol through a multi-step enzymatic cascade occurring within the zona glomerulosa of the adrenal cortex. Its biosynthesis is rate-limited by CYP11B2, commonly designated as aldosterone synthase (AS), which sequentially hydroxylates 11-deoxycorticosterone to yield corticosterone, followed by 18-hydroxycorticosterone, and finally catalyzes oxidation to the biologically active aldosterone. A critical pharmacological challenge in targeting CYP11B2 stems from its greater than 90% structural homology with CYP11B1, the enzyme governing cortisol biosynthesis through 11β-hydroxylation [2] (Figure 1). The close homology between these enzymes has posed a major clinical challenge, as it has historically hindered the development of selective ASIs that target CYP11B2 without affecting cortisol synthesis. Aldosterone is not synthetized solely in the adrenal cortex, but de novo synthesis can occur outside the adrenal glands including the kidneys. Local production of aldosterone has been demonstrated in renal mesangial cells and podocytes. Intrarenal generation and concentration of aldosterone are physiologically relevant and eventually more important than systemic levels for kidney injury among individuals with diabetes [3].

Aldosterone biosynthesis is principally governed by three physiological stimuli: angiotensin 2 (Ang II), extracellular potassium concentration, and adrenocorticotropic hormone (ACTH). Ang II originates from the proteolytic cleavage of angiotensinogen, first cleaved by renin to yield angiotensin I, which is subsequently converted to Ang II by angiotensin-converting enzyme. Within the adrenal cortex, Ang II engages angiotensin type 1 (AT1) receptors expressed on zona glomerulosa cells, triggering calcium-dependent signaling pathways that stimulate aldosterone production. Serum potassium directly influences aldosterone secretion by inducing membrane depolarization in the adrenal cortical cells. Additionally, ACTH modulates aldosterone production via melanocortin 2 receptor-mediated signaling, also involving intracellular calcium dynamics. In addition to these classical regulators, several metabolic factors relevant to diabetes contribute to increased aldosterone production. For instance, hyperglycemia has been shown to upregulate CYP11B2 transcription and mRNA expression in adrenal cells but also locally in the kidneys [4]. Leptin, secreted by adipocytes, is also a direct regulator of AS by binding to leptin receptors on adrenal cells in the zona glomerulosa [5]. Aldosterone excess has also been demonstrated to suppress pancreatic insulin secretion and attenuate insulin-mediated glucose uptake in skeletal muscle, which in turn elevates glucose levels further promoting aldosterone production. Collectively, these interactions suggest a deleterious feedback loop in which metabolic dysfunction potentiates excess aldosterone production and hence worsens glycemic control.

## 3. Mechanism of Aldosterone Action

**Figure 2:**
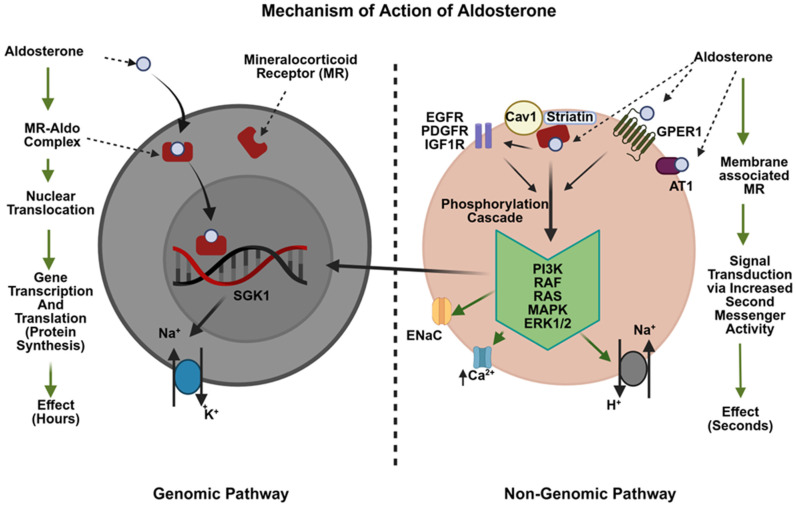
Genomic and non-genomic actions of aldosterone. AT1: angiotensin II type 1 receptor; Cav1: caveolin-1; EGFR: epidermal growth factor receptor; ENaC: epithelial sodium channel; ERK1/2: extracellular signal-regulated kinase; GPER1: G protein-coupled estrogen receptor 1; IGF1R: insulin-like growth factor receptor-1; MAPK: mitogen-activated protein kinase; MR: mineralocorticoid receptor: PDGFR: platelet-derived growth factor receptor; PI3K phosphatidylinositol 3-kinase; SGK1: serum and glucocorticoid-regulated kinase 1.

Aldosterone mediates its biologic effects through two principal signaling mechanisms: genomic and non-genomic pathways. The genomic pathway, often referred to as the classic or delayed pathway, is initiated when aldosterone diffuses across the plasma membrane and binds to the intracellular mineralocorticoid receptor (MR), a ligand-dependent nuclear transcription factor located within the cytoplasm. Following receptor binding, the aldosterone–MR complex translocates into the nucleus where it interacts with hormone-responsive DNA elements, resulting in transcriptional regulation of multiple target genes (Figure 2). Because this process depends on both gene transcription and protein synthesis, the downstream physiological effects generally develop over several hours. Although MR expression was originally identified in epithelial cells of the distal nephron, particularly the distal convoluted tubule and collecting duct, subsequent investigations have demonstrated widespread MR distribution in non-epithelial renal cells, as well as in cardiovascular, vascular, and neural tissues [6].

Among the genes activated by aldosterone signaling, serum- and glucocorticoid-inducible kinase 1 (SGK1) has emerged as a major downstream effector involved in sodium handling, inflammation, and fibrotic remodeling. Growing evidence indicates that activation of the aldosterone-SGK1 pathway contributes to the progression of kidney injury [7]. Experimental studies in mesangial cells have demonstrated that aldosterone increases SGK1 expression and activity, which in turn enhances NF-κappa B mediated transcriptional pathways and stimulates the production of inflammatory and fibrotic mediators such as intercellular adhesion molecule-1 (ICAM-1) and connective tissue growth factor (CTGF) [7,8,9,10]. Consistent findings have emerged from experimental animal models, in which sustained aldosterone exposure produced marked upregulation of renal SGK1 expression and profibrotic markers, accompanied by glomerulosclerosis, hypertension, and inflammatory injury. Taken together, these observations establish aldosterone-driven genomic signaling as a mechanistically significant contributor to the initiation and perpetuation of renal inflammatory and fibrotic processes.

The non-genomic, or rapid, pathway of aldosterone signaling involves membrane-associated receptor mechanisms that initiate intracellular signaling within seconds to minutes. Although some rapid effects appear to be independent of the classical MR, accumulating evidence suggests that many are at least partially mediated through membrane-associated MR signaling complexes. Unlike traditional transmembrane receptors, the MR lacks a transmembrane domain and is instead localized along the cytosolic aspect of the cell membrane. Scaffold proteins such as caveolin-1 and striatin facilitate interactions between the MR and several membrane-bound receptor tyrosine kinases, including epidermal growth factor receptor (EGFR), platelet-derived growth factor receptor (PDGFR), and insulin-like growth factor receptor-1 (IGF1R) [11,12]. Activation of these receptor complexes triggers multiple downstream phosphorylation cascades involving phosphatidylinositol 3-kinase (PI3K), protein kinase C (PKC), Ras/Raf, mitogen-activated protein kinase (MAPK), extracellular signal-regulated kinase (ERK1/2), and MEK signaling pathways [13,14,15]. These signaling events rapidly modulate cellular ion transport and vascular responses through increased intracellular calcium signaling, activation of Na^+^/H^+^ exchangers, stimulation of Na^+^/K^+^-ATPase activity, and enhanced epithelial sodium channel (ENaC) function [14,16].

In addition to membrane-associated MR signaling, other cell surface receptors, particularly the G protein-coupled estrogen receptor (GPER1), have also been proposed as mediators of aldosterone-induced rapid signaling [17]. Activation of GPER1 has been linked to downstream stimulation of cyclic AMP (cAMP), PI3K/Akt, ERK1/2, and intracellular calcium-dependent signaling pathways, contributing to rapid alterations in endothelial function, vascular tone, oxidative stress, and inflammatory responses [17,18]. Collectively, these membrane-associated signaling networks produce rapid physiological effects that occur independently of transcriptional regulation while also interacting closely with genomic MR pathways [13,14]. Increasing evidence suggests substantial crosstalk between genomic and non-genomic signaling, whereby rapid kinase activation may subsequently amplify transcriptional responses and contribute to progression of kidney injury [18,19] (Figure 2).

## 4. Role of Renin–Angiotensin–Aldosterone System in Diabetic Kidney Disease

Activation of RAAS plays a key role in the development of DKD. However, DKD is paradoxically a state of low systemic renin, ang II, and aldosterone [20]. While there is no significant activation of systemic RAAS among individuals with DKD, the local intrarenal RAAS upregulation primarily contributes to the pathogenesis of DKD. Local levels of aldosterone which exert a paracrine effect can be higher in the kidneys as compared to the systemic levels, which are usually low in DKD.

Within the kidney, aldosterone serves as a well-established physiological function governing sodium and potassium homeostasis through a complex neuroendocrine system responsible for water, electrolyte, and blood pressure control. The classic renal action of aldosterone is to enhance the apical membrane expression of the epithelial sodium channel in principal cells of the distal nephron, driving net sodium reabsorption and obligatory fluid retention. When aldosterone binds to the MR, it alters gene transcription increasing glucocorticoid-induced kinase 1 leading to altered expression of ENaC. However, aldosterone exerts direct pathophysiological effects within the kidney under conditions associated with local RAAS activation, independently driving glomerular injury, interstitial inflammatory infiltration, and progressive fibrotic remodeling through mechanisms that operate independently of circulating ang II.

The action of aldosterone is not restricted to epithelial cells in the kidneys. Aldosterone also affects endothelial cells, vascular smooth muscle cells, podocytes, mesangial cells, immune cells, and fibroblasts. In endothelial cells, aldosterone promotes dysfunction by inducing reactive oxygen species and damaging the glomerular endothelial glycocalyx, thereby contributing to albuminuria. Aldosterone further promotes vascular inflammation by stimulating macrophage colony stimulating factor (M-CSF) secretion from vascular smooth muscle cells, inducing their phenotypic transformation toward a macrophage-like state and facilitating their participation in the formation of inflammatory vascular lesions [21]. Aldosterone also induces reactive oxygen species (ROS) in podocytes via Ras-related C3 botulinum toxin substrate 1 (Rac1) activation leading to oxidative damage of cellular proteins and lipids leading resulting in podocyte dysfunction, particularly through disruption of cytoskeletal organization [22]. In mesangial cells, aldosterone promotes proliferation while also inducing cellular injury and apoptosis, leading to mesangial expansion [23,24]. It further enhances inflammatory signaling by upregulating adhesion molecules such as intercellular adhesion molecule 1 (ICAM-1) and vascular cell adhesion molecule-1 (VCAM-1), promoting the recruitment of monocytes and macrophages and amplifying inflammation-mediated injury [25]. Within renal tubular epithelial cells, aldosterone drives phenotypic dedifferentiation through induction of epithelial-to-mesenchymal transition, generating activated fibroblasts that directly contribute to interstitial collagen deposition. Concurrently, aldosterone upregulates transforming growth factor beta 1 (TGF-β1) ligand expression, establishing a paracrine profibrotic loop that sustains fibroblast proliferation and accelerates extracellular matrix accumulation independently of continued epithelial cell trans-differentiation [26].

Beyond the classical genomic and non-genomic signaling, aldosterone also activates the NLRP3 inflammasome, a multiprotein cytoplasmic complex comprising NLRP3, the adaptor protein ASC, and pro-caspase-1 in renal tubular cells and podocytes through a MR-dependent mechanism. Aldosterone stimulates mitochondrial ROS generation via MR activation, which serves as the primary danger signal triggering NLRP3 assembly and activation. Activated NLRP3 recruits and cleaves pro-caspase-1, generating active caspase-1 that proteolytically matures pro-IL-1β and pro-IL-18 into their biologically active forms, triggering a self-sustaining inflammatory amplification loop. The MR-dependence of this pathway was confirmed experimentally by the dose-dependent suppression of NLRP3 inflammasome activity achieved with eplerenone, which distinguished aldosterone-driven NLRP3 activation from a generalized cellular stress response [27].

Causal confirmation of this pathway was provided by genetic studies in which NLRP3-deficient mice subjected to aldosterone infusion exhibited markedly diminished renal IL-1β and IL-18 processing, reduced tubular epithelial apoptosis, and preservation of normal epithelial cell morphology, collectively establishing that MR-dependent NLRP3 activation is an obligatory mediator of aldosterone-induced tubular injury rather than an epiphenomenon of the inflammatory response [27]. This mechanism is particularly relevant in DKD, where local aldosterone excess in the kidney operates even in the setting of suppressed systemic RAAS, podocytes are already vulnerable to oxidative stress, and hemodynamic injury is further damaged by aldosterone-driven NLRP3 activation, leading to cytoskeletal disruption, foot process effacement, and progressive albuminuria [28]. Collectively, the aldosterone-MR-mitochondrial ROS-NLRP3-IL-1β axis represents an inflammation amplification pathway that operates in parallel with and independently of classical MR transcriptional signaling, contributing to the inflammatory milieu that characterizes DKD progression and that is not fully addressed by conventional RAAS blockade alone.

Additionally, Aldosterone-induced mitochondrial dysfunction represents a mechanistically distinct contributor to renal injury in DKD that extends beyond the oxidative stress pathways described above. Aldosterone suppresses the activity of peroxisome proliferator-activated receptor-gamma coactivator 1-alpha (PGC-1α), the master transcriptional regulator of mitochondrial biogenesis in podocytes, resulting in reduced mitochondrial mass, impaired respiratory chain function, and deficient ATP generation. When PGC-1α activity is experimentally preserved or restored, aldosterone-driven mitochondrial impairment and podocyte loss are substantially attenuated both in vitro and in vivo, confirming that PGC-1α downregulation is a causally relevant and therapeutically targetable mechanism rather than an epiphenomenon of aldosterone-mediated injury [29]. The downstream consequences of this mitochondrial impairment include disruption of mitochondrial membrane potential, augmented superoxide production from the electron transport chain, lipid accumulation within tubular and podocyte compartments, and activation of intrinsic apoptotic pathways, all histological and functional features consistently observed in DKD. Critically, this mechanism operates in conjunction with the NLRP3 pathway described above: mitochondrial ROS generated downstream of aldosterone-mediated PGC-1α suppression serves simultaneously as a trigger for NLRP3 inflammasome assembly, creating a mechanistically integrated aldosterone-mitochondria-NLRP3 injury axis that amplifies both inflammatory and metabolic damage in the diabetic kidney [27,29].

The profibrotic actions of aldosterone in DKD are substantially mediated through the TGF-β/Smad signaling axis. Aldosterone upregulates TGF-β1 ligand expression in renal tubular epithelial cells and fibroblasts [26], initiating a downstream signaling cascade in which TGF-β1 binds its cognate receptors and drives phosphorylation of Smad2 and Smad3. Following phosphorylation, the activated Smad2/3 complex recruits Smad4 as an obligatory co-mediator, and the resultant heterotrimeric complex undergoes nuclear translocation where it engages promoter elements of genes encoding structural extracellular matrix proteins, including fibronectin, collagen I, collagen IV, and laminin, while simultaneously suppressing matrix metalloproteinase activity, disrupting the equilibrium between matrix deposition and degradation in favor of progressive interstitial accumulation [30]. In parallel, phosphorylated Smad3 orchestrates the phenotypic reprogramming of tubular epithelial cells toward a mesenchymal state, generating activated myofibroblasts that amplify interstitial collagen deposition and perpetuate the fibrotic program independently of continued aldosterone stimulation. In the context of DKD, this TGF-β/Smad cascade is particularly injurious because it operates in a glucotoxic milieu where high glucose independently primes Smad3 phosphorylation, meaning that aldosterone-driven TGF-β1 upregulation activates a signaling axis that is already sensitized by the diabetic microenvironment, producing a synergistic fibrotic response that exceeds what either stimulus could generate alone [30]. This cooperative mechanism contributes to the accelerated tubulointerstitial fibrosis and glomerulosclerosis that distinguish DKD from non-diabetic CKD and explains in part why conventional RAAS blockade, which reduces but does not eliminate aldosterone-driven TGF-β1 induction, achieves only partial attenuation of fibrotic progression.

### Crosstalk with SGLT2 Inhibitor Pathways at the Molecular Level

The molecular mechanisms by which aldosterone drives DKD injury share several convergent targets with the pathways modulated by SGLT2 inhibitors, creating a compelling biological rationale for their combined use in DKD that extends beyond their independent hemodynamic and metabolic benefits. SGLT2 inhibitors suppress NLRP3 inflammasome activation in the kidney through a mechanism involving upregulation of the metabolite itaconate, thereby attenuating the caspase-1-dependent generation of IL-1β and IL-18 that drives tubular inflammation and fibrosis [31]. Because aldosterone independently activates this same NLRP3 pathway through MR-dependent mitochondrial ROS generation, the convergence of both stimuli on NLRP3 in the diabetic kidney suggests that SGLT2 inhibitors may partially counteract aldosterone-driven renal inflammation, and conversely, that residual NLRP3 activity persisting despite SGLT2 inhibition may reflect ongoing aldosterone excess that is not addressed by SGLT2 blockade alone. At the mitochondrial level, SGLT2 inhibitors activate AMPK and upregulate PGC-1α, the same transcriptional coactivator that aldosterone suppresses in podocytes [29], restoring mitochondrial biogenesis and oxidative phosphorylation from the opposite direction. This mechanistic opposition regarding shared molecular targets, NLRP3 inflammasome assembly, mitochondrial PGC-1α expression, and downstream oxidative stress provides a molecular framework for understanding why SGLT2 inhibitors and aldosterone-targeted therapies produce complementary renoprotection in DKD and furnishes the mechanistic rationale for ongoing combination trials of second-generation ASIs with empagliflozin and dapagliflozin.

## 5. Pharmacotherapy to Modulate Aldosterone

**Figure 3:**
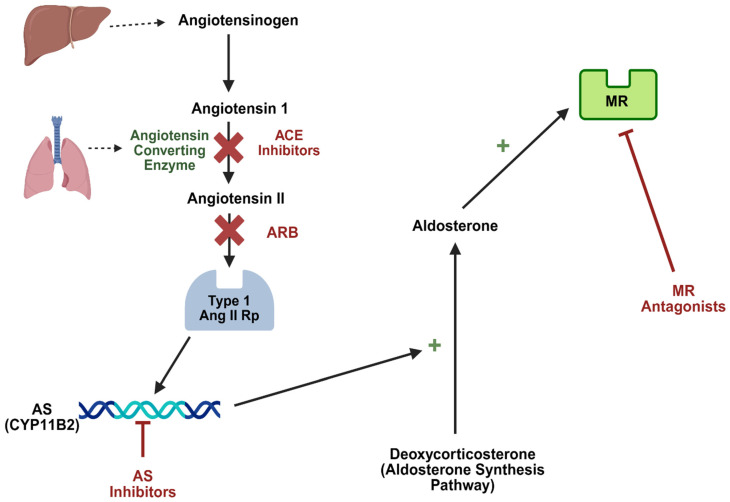
Therapeutic modulation of RAAS. ACE inhibitors: angiotensin-converting enzyme inhibitors; Ang II: angiotensin II; ARB: angiotensin receptor blocker; AS: aldosterone synthase; MR: mineralocorticoid receptor; RAAS: renin–angiotensin–aldosterone system.

The mainstay of treatment for dysregulation of aldosterone is currently based on the use of three different classes of drugs which have their use supported by strong clinical evidence in relation to their renoprotective effects among individuals with DKD (Figure 3).

### 5.1. ACE Inhibitors and Angiotensin Receptor Blockers

ACE inhibitors and ARBs were the first drugs to show a renoprotective effect among patients with DKD. ACE inhibitors are competitive inhibitors of the angiotensin converting enzyme blocking the conversion of angiotensin I to Ang II. Meanwhile, ARBs selectively inhibit the binding of Ang II to the AT1 receptor. The benefits of ACE inhibitors and ARBs are secondary to reduction in systemic and intraglomerular blood pressure and decreased albuminuria mediated by a reduction in the levels or by blocking the effect of Ang II which is the key mediator of aldosterone production [32]. Landmark trials conducted three decades ago established that RAAS blockade with ACE inhibitors or ARBs conferred renoprotective benefits independent of their antihypertensive effects, with ACE inhibitors reducing the risk of renal failure by 39% and ARBs by 18% relative to placebo [33].

### 5.2. Mineralocorticoid Receptor Antagonists

MRAs exert their pharmacological effect by competitively inhibiting the ligand-binding domain of the mineralocorticoid receptor, thereby displacing aldosterone and blocking the receptor activation and downstream genomic signaling that drives renal and cardiovascular injury. While steroidal MRAs have an established role in cardiovascular therapeutics, their applicability to CKD populations has been substantially constrained by the clinically significant risk of hyperkalemia, a consequence of their mechanism-based inhibition of renal potassium secretion in patients with already compromised tubular function. This has led to the development of non-steroidal MRAs (nsMRAs), which offer greater receptor selectivity and in theory an improved safety profile. Finerenone, an nsMRA, occupies a distinct mechanistic role among renoprotective therapies. Its effects on intraglomerular hemodynamics are relatively modest compared with those of RAAS inhibitors and SGLT2 inhibitors, consistent with the smaller acute decline in estimated glomerular filtration rate (eGFR) observed in clinical trials. These hemodynamic effects are thought to be mediated in part through modulation of distal tubular sodium handling and enhancement of tubuloglomerular feedback, resulting in a modest reduction in intraglomerular pressure [34].

The renoprotective benefits of finerenone extend beyond blood pressure reduction and glomerular hemodynamic improvement, with accumulating evidence implicating direct anti-inflammatory and anti-fibrotic mechanisms as additional contributors to its organ-protective effects. MR overactivation in renal cells promotes the expression of profibrotic and proinflammatory mediators, contributing to structural damage within the glomerular filtration barrier and a reduction in the ultrafiltration coefficient. By inhibiting these pathways, finerenone addresses mechanisms of kidney injury that are not fully targeted by therapies acting primarily on arteriolar tone. Clinically, finerenone represents a major advancement in aldosterone-targeted therapy and is currently the only nsMRA approved for use in DKD. The FIDELIO-DKD trial demonstrated that finerenone reduced the composite risk of kidney disease progression by 18% relative to placebo in patients with CKD and type 2 diabetes, establishing its role as an evidence-based addition to contemporary DKD management alongside RAAS inhibition and SGLT2 inhibitors [35].

### 5.3. Aldosterone Breakthrough Mechanism

Aldosterone breakthrough refers to the phenomenon in which ACE inhibitors or ARBs fail to sustain suppression of aldosterone levels. Despite initial suppression of plasma aldosterone following RAAS inhibitor initiation, a paradoxical resurgence to near-baseline concentrations develops in approximately 30% of patients during sustained therapy, a phenomenon designated as aldosterone breakthrough that fundamentally limits the durability of RAAS blockade as an aldosterone-suppressing strategy [36,37]. This phenomenon reflects the contribution of ACE independent pathways of Ang II generation (e.g., chymase), as well as potassium and ACTH-mediated stimulation of aldosterone synthesis. As a result, persistent or rising aldosterone levels may continue to drive MR mediated inflammation and fibrosis despite ACE inhibitor or ARB therapy. MRAs have been proposed to mitigate aldosterone breakthrough by directly blocking MR activation, thereby attenuating the downstream effects of persistently elevated or residual aldosterone despite ACE inhibition or ARB therapy. However, MRA therapy induces a compensatory increase in plasma renin and aldosterone concentrations, which may partially overcome receptor blockade and permit continued MR-independent effects of aldosterone. Consequently, current RAAS-targeted therapies, including ACE inhibitors, ARBs, and MRAs, may result in incomplete suppression of aldosterone activity, allowing the progression of DKD and necessitating more therapeutic interventions.

### 5.4. Aldosterone Synthase Inhibitors

Aldosterone synthase inhibitors (ASIs) reduce the production of aldosterone directly by inhibiting AS. These drugs offer the theoretical benefit of avoiding the aldosterone breakthrough mechanism. Osilodrostat was the first ASI developed and examined in clinical studies. Early trials demonstrated that AS inhibition was a feasible therapeutic approach for conditions such as hypertension and primary aldosteronism. However, its limited selectivity for CYP11B2 resulted in inhibition of CYP11B1, thereby interfering with cortisol synthesis and precluding its further development for cardiovascular and kidney diseases [38]. To overcome the selectivity limitations of first-generation agents, three second-generation ASIs with substantially improved CYP11B2 specificity—baxdrostat, lorundrostat, and vicadrostat—have advanced into clinical investigation for the treatment of CKD. Several phase II and III trials include individuals with CKD and diabetes, making patients with DKD, the intersection between those two conditions, a key subgroup of interest. Recently completed and ongoing clinical trials focusing on individuals with CKD are summarized on Table 1.

### 5.5. Baxdrostat

The results of a randomized, double-blinded, placebo-controlled, phase II trial for the use of baxdrostat in patients with CKD and uncontrolled hypertension was recently published [39]. Eligible participants were treated with an ACE inhibitor or ARB and had a urine albumin–creatinine ratio (UACR) of ≥100 mg/g. Participants were randomized to baxdrostat low-dose, high-dose, or placebo for 26 weeks. The primary endpoint was change from baseline in mean systolic blood pressure (BP) at week 26 in the baxdrostat pooled treatment group versus placebo. Among individuals included, 80% had type 2 diabetes and the median UACR was 714 mg/g. In total, 195 participants were randomized. Beyond its blood pressure effects, the trial demonstrated that baxdrostat produced a mean reduction in systolic blood pressure of 8 mmHg from baseline to week 26, alongside a 55.2% decrease in UACR relative to placebo over the same period. The safety and efficacy of baxdrostat combined with dapagliflozin in slowing CKD progression is the subject of three additional ongoing trials [40,41,42].

### 5.6. Lorundrostat

A single clinical trial was completed but not peer-reviewed yet [43]. This trial employed a randomized, placebo-controlled crossover design to assess whether lorundrostat 25 mg, added to background SGLT2 inhibitor and ACE inhibitor or ARB therapy, improved renal and hemodynamic outcomes in the enrolled 59 patients with CKD. The study population was predominantly diabetic (76%) with preserved kidney function (eGFR ≥ 30 mL/min/1.73 m^2^) and significant albuminuria (200–5000 mg/g). At the end of the treatment period, lorundrostat-treated participants achieved a 9.25 mmHg reduction in systolic blood pressure and a 30.5% decrease in UACR, compared with reductions of 1.76 mmHg and 6.60% in the placebo arm, representing clinically meaningful between-group differences across both endpoints.

### 5.7. Vicadrostat

The results of a randomized controlled phase II clinical trial evaluating the efficacy and safety of vicadrostat combined with empagliflozin was recently published. Enrollees were individuals aged 18 years or older with an eGFR between 30 and 90 mL/min/1.73 m^2^, a UACR between 200 and 5000 mg/g, and already on maximized RAAS inhibition with an ACE inhibitor or ARB. Using a two-step randomization approach, participants first received empagliflozin or placebo during an 8-week run-in phase, after which they were assigned to once-daily vicadrostat at 3 mg, 10 mg, or 20 mg, or placebo, for 14 weeks. The primary endpoint was the change in UACR from baseline at the end of treatment. Of the 714 run-in participants, 586 were randomly assigned to receive vicadrostat or placebo. At baseline, mean baseline eGFR was 51.9 mL/min/1.73 m^2^ and median UACR was 426 mg/g. At week 14, placebo-treated participants experienced a 3% reduction in UACR from baseline, whereas those receiving vicadrostat monotherapy achieved substantially greater reductions in a dose-dependent pattern, 22%, 39%, and 37% with the 3 mg, 10 mg, and 20 mg doses respectively. Addition of vicadrostat to empagliflozin produced UACR reductions of comparable magnitude to those observed with vicadrostat alone [44]. Two other trials are currently ongoing and each of these trials is designed to evaluate whether the addition of vicadrostat to empagliflozin confers superior safety and efficacy compared with empagliflozin monotherapy in patients with CKD at elevated risk of disease progression [45,46].

### 5.8. Limitations of Current Evidence About Aldosterone Synthase Inhibitors in Chronic Kidney Disease

Overall, up to this point, clinical trials examining ASIs among individuals with CKD have included small sample sizes and they have examined soft outcomes such as reductions in blood pressure and UACR during short periods of follow up, without examining hard endpoints such as sustained eGFR decline, kidney failure, or cardiovascular death. Larger clinical trials that will include thousands of participants and examine hard outcomes are still ongoing. Trials such as BaxDuo-Pacific and EASi-KIDNEY will add robust evidence about the efficacy of ASIs but also adverse events such as hyperkalemia and adrenal insufficiency. Those trials might still lack the ability to answer questions about the use of ASIs among those with advanced CKD since the inclusion criteria requires an eGFR above 20–30 mL/min/1.73 m^2^. For all trials, ASIs are being examined among individuals on a background therapy based on ACE inhibitors/ARBs and ASIs are being added on top of SGLT2 inhibitors in certain trials. Therefore, those trials will offer information about the combined use of those drug classes although, to our knowledge, no studies are currently planned comparing ASIs and MRAs. Additionally, standardized protocols for cortisol monitoring and management of adrenal insufficiency risk during physiological stress, such as sepsis, surgery, or volume depletion, have not yet been established for any second-generation ASI, representing an important pharmacovigilance gap as these agents advance toward regulatory approval.

## 6. Comparison of Mineralocorticoid Receptor Antagonists and Aldosterone Synthase Inhibitors

MRAs and ASIs both target aldosterone but diverge substantially in their mechanism of action, efficacy profile, and safety considerations. While MRAs block the mineralocorticoid receptor downstream, ASIs interrupt aldosterone production itself, a distinction with potentially meaningful clinical implications. Direct comparisons between steroidal and nsMRAs in DKD remain methodologically constrained by the paucity of head-to-head trials. An umbrella review of meta-analyses demonstrated that the preponderance of evidence has evaluated nsMRAs, which have consistently demonstrated reductions in kidney events, UACR, and risk of dialysis initiation compared with placebo. At the same time, nsMRAs were also associated with a two to fourfold increase in hyperkalemia risk relative to placebo [47]. The direct head-to-head comparison between MRAs and ASIs is not yet possible, as no randomized controlled trials have evaluated these drug classes against each other. Available data are therefore limited to indirect comparisons. A recent meta-analysis of clinical trials examining second-generation ASIs in hypertension management reported an odds ratio of 7.1 for hyperkalemia compared with placebo without a significant difference in adrenal insufficiency risk [48]. Critically, those trials enrolled predominantly patients with preserved renal function, limiting the applicability of these findings to CKD populations in whom aldosterone dysregulation and baseline hyperkalemia risk are substantially greater. Prospective evaluation of ASI safety specifically in advanced CKD and DKD populations is therefore needed before routine clinical adoption can be recommended. Taken together, the available evidence suggests that both drug classes carry meaningful hyperkalemia risk through convergent mechanisms, interference with aldosterone-mediated potassium secretion, regardless of whether aldosterone is blocked at the receptor level or at the level of synthesis. This shared liability underscores the importance of regular electrolyte surveillance and judicious patient selection when considering either agent in clinical practice, particularly among CKD patients already at an elevated risk for hyperkalemia. The risk of adrenal insufficiency meanwhile with second-generation ASIs does not seem clinically relevant.

## 7. Future Directions

Aldosterone has long been viewed through the narrow lens of blood pressure and volume regulation; however, this paradigm is increasingly inadequate. A growing body of evidence positions aldosterone as a central driver in the development of DKD. Despite the widespread use of RAAS inhibitors, including ACE inhibitors, ARBs, and MRAs, residual kidney risk remains substantial. The phenomenon of aldosterone breakthrough, along with compensatory increases in circulating aldosterone, underscores a fundamental limitation of current strategies: incomplete and often transient suppression of aldosterone activity. ASIs challenge this paradigm by targeting aldosterone at its source. By reducing aldosterone production, ASIs have the potential to mitigate both MR-dependent and MR-independent effects that persist despite conventional therapy. Unlike MRAs, which block receptor activation, ASIs reduce circulating aldosterone levels, offering a more comprehensive approach to aldosterone inhibition. If ongoing clinical trials confirm their efficacy and safety, ASIs may prompt a reappraisal of current treatment algorithms, shifting the field from receptor-level blockade toward upstream hormonal suppression. In the contemporary management of DKD, ACE inhibitors, ARBs, and non-steroidal MRAs represent foundational therapies, significantly reducing the risk of progressive kidney disease. The emergence of ASIs introduces a new dimension to RAAS modulation, raising important questions regarding their optimal positioning within existing treatment paradigms, including whether they should be used sequentially, in combination, or as alternatives to MRAs in selected patient populations. If validated by ongoing trials, ASIs may redefine aldosterone-targeted therapy in DKD, positioning upstream hormonal suppression alongside established hemodynamic and receptor-based strategies. In this evolving landscape, ASIs may not merely be an addition to current therapies but could represent a potential inflection point in the management of DKD.

## Figures and Tables

**Table 1:** Completed and ongoing clinical trials with aldosterone synthase inhibitors among individuals with chronic kidney disease.

Study Name	Design	Status	Population	Sample Size	Endpoint	Start Date	Completion
**Baxdrostat**							
FigHTN (NCT05432167) [39]	Randomized, double-blind, placebo-controlled Phase II	Completed	Participants aged ≥18 years treated with ACEi/ARB with an eGFR of 25–75 mL/min/1.73 m^2^, UACR of ≥100 mg/g, and SBP ≥ 140 mmHg without diabetes or ≥130 mmHg with type 2 diabetes	195	Primary: change in mean SBP from baseline to week 26 Exploratory: change from baseline UACR	29 April 2022	2 May 2024
NCT06268873 [40]	Randomized, double-blind, placebo-controlled Phase III	Active but not recruiting	Eligible patients aged ≥18 years treated with ACEi/ARB with an eGFR 30–90 mL/min/1.73 m^2^, UACR > 200 and <5000 mg/g and history of HTN	2554 (estimated)	Primary: changes in eGFR from baseline to post-treatment Secondary: change from baseline in UACR	29 March 2024	24 February 2028
BaxDuo-Baltic (NCT07222917) [41]	Randomized, double-blind, placebo-controlled Phase IIb	Recruiting	Eligible patients aged ≥18 years treated with ACEi/ARB with eGFR 30–90 mL/min/1.73 m^2^, UACR of 200–5000 mg/g, and history of HTN	218 (estimated)	Primary: change in UACR from baseline to week 12	5 December 2025	24 May 2027
BaxDuo-Pacific (NCT06742723) [42]	Randomized, double-blind, placebo-controlled Phase III	Recruiting	Eligible patients aged ≥18 years treated with ACEi/ARB with eGFR 30–75 mL/min/1.73 m^2^, UACR of 30–5000 mg/g, and history of HTN	5000 (estimated)	Primary: time to first occurrence of kidney disease progression or CV events	3 March 2025	18 December 2029
**Lorundrostat**							
Explore-CKD (NCT06150924) [43]	Randomized, double-blind, placebo-controlled, crossover Phase II	Completed	Participants aged ≥18 years treated with an ACEi/ARB with eGFR of ≥30 mL/min/1.73 m^2^, UACR of 200–5000 mg/g, and SBP of 135–180 mmHg	59	Primary: change in SBP from baseline to week 4 Secondary: change in 24 h urine albumin at week 4	14 December 2023	27 February 2025
**Vicadrostat**							
NCT05182840 [44]	Randomized, double-blind, placebo-controlled Phase II	Completed	Participants aged ≥18 years treated with ACEi/ARB with eGFR 20–60 mL/min/1.73 m^2^, UACR of 200–5000 mg/g	714	Primary: change in UACR from baseline to week 14	11 January 2022	19 June 2023
NCT06926660 [45]	Randomized, double-blind, placebo-controlled Phase II	Active, not recruiting	Eligible patients aged ≥18 years treated with ACEi/ARB with eGFR 30–90 mL/min/1.73 m^2^ irrespective of UACR	492	Primary: change in eGFR from baseline to week 16 Secondary: change in UACR from baseline to week 16	18 July 2025	20 August 2026
EASi-KIDNEY (NCT06531824) [46]	Randomized, double-blind, placebo-controlled Phase III	Recruiting	Eligible patients aged ≥18 years with eGFR 20–45 or 45–90 mL/min/1.73 m^2^ with UACR ≥ 200 mg/g	11,000 (estimated)	Primary: time to first occurrence of the primary composite outcome of kidney disease progression, hospitalization for heart failure or cardiovascular death	13 August 2024	30 August 2028

ACEi: angiotensin-converting enzyme inhibitor; ARB: angiotensin receptor blocker; eGFR: estimated glomerular filtration rate; HTN: hypertension; CKD: chronic kidney disease; NCT: National Clinical Trial Number; SBP: systolic blood pressure; UACR: urine albumin–creatinine ratio.

## Data Availability

No new data were created or analyzed in this study. Data sharing is not applicable to this article.

## Abbreviations

ACE inhibitors	angiotensin-converting enzyme inhibitors
ACTH	adrenocorticotropic hormone
Ang II	angiotensin II
ARB	angiotensin receptor blocker
AS	aldosterone synthase
ASI	aldosterone synthase inhibitor
AT1 receptor	angiotensin type 1 receptor
BP	blood pressure
cAMP	cyclic AMP
CKD	chronic kidney disease
CTGF	connective tissue growth factor
DKD	diabetic kidney disease
eGFR	estimated glomerular filtration rate
EGFR	epidermal growth factor receptor
ENaC	epithelial sodium channel
ERK1/2	extracellular signal-regulated kinase
GPER1	G protein-coupled estrogen receptor
ICAM-1	intercellular adhesion molecule-1
IGF1R	insulin-like growth factor receptor-1
MAPK	mitogen-activated protein kinase
M-CSF	macrophage colony stimulating factor
MR	mineralocorticoid receptor
MRA	mineralocorticoid receptor antagonist
nsMRA	non-steroidal mineralocorticoid receptor antagonist
PDGFR	platelet-derived growth factor receptor
PGC-1α	peroxisome proliferator-activated receptor-gamma coactivator 1-alpha
PI3K	phosphatidylinositol 3-kinase
PKC	protein kinase C
RAAS	renin–angiotensin–aldosterone system
Rac1	Ras-related C3 botulinum toxin substrate 1
ROS	reactive oxygen species
SGK1	serum- and glucocorticoid-inducible kinase 1
TGF-β	transforming growth factor-β
UACR	urine albumin–creatinine ratio
VCAM-1	vascular cell adhesion molecule-1

## References

[1] Ong K.L., Stafford L.K., McLaughlin S.A., Boyko E.J., Vollset S.E., Smith A.E., Dalton B.E., Duprey J., Cruz J.A., Hagins H. (2023). Global, regional, and national burden of diabetes from 1990 to 2021, with projections of prevalence to 2050: A systematic analysis for the Global Burden of Disease Study 2021. Lancet.

[2] Bureik M., Lisurek M., Bernhardt R. (2002). The human steroid hydroxylases CYP1B1 and CYP11B2. Biol. Chem..

[3] Taves M.D., Gomez-Sanchez C.E., Soma K.K. (2011). Extra-adrenal glucocorticoids and mineralocorticoids: Evidence for local synthesis, regulation, and function. Am. J. Physiol. Endocrinol. Metab..

[4] Shimada H., Kogure N., Noro E., Kudo M., Sugawara K., Sato I., Shimizu K., Kobayashi M., Suzuki D., Parvin R. (2017). High glucose stimulates expression of aldosterone synthase (CYP11B2) and secretion of aldosterone in human adrenal cells. FEBS Open Bio.

[5] Huby A.C., Antonova G., Groenendyk J., Gomez-Sanchez C.E., Bollag W.B., Filosa J.A., Belin de Chantemèle E.L. (2015). Adipocyte-Derived Hormone Leptin Is a Direct Regulator of Aldosterone Secretion, Which Promotes Endothelial Dysfunction and Cardiac Fibrosis. Circulation.

[6] Johnston J.G., Welch A.K., Cain B.D., Sayeski P.P., Gumz M.L., Wingo C.S. (2023). Aldosterone: Renal Action and Physiological Effects. Compr. Physiol..

[7] Terada Y., Kuwana H., Kobayashi T., Okado T., Suzuki N., Yoshimoto T., Hirata Y., Sasaki S. (2008). Aldosterone-stimulated SGK1 activity mediates profibrotic signaling in the mesangium. J. Am. Soc. Nephrol..

[8] Moussad E.E., Brigstock D.R. (2000). Connective tissue growth factor: What’s in a name?. Mol. Genet. Metab..

[9] Wang S., Denichilo M., Brubaker C., Hirschberg R. (2001). Connective tissue growth factor in tubulointerstitial injury of diabetic nephropathy. Kidney Int..

[10] Ito Y., Aten J., Bende R.J., Oemar B.S., Rabelink T.J., Weening J.J., Goldschmeding R. (1998). Expression of connective tissue growth factor in human renal fibrosis. Kidney Int..

[11] Baudrand R., Pojoga L.H., Romero J.R., Williams G.H. (2014). Aldosterone’s mechanism of action: Roles of lysine-specific demethylase 1, caveolin and striatin. Curr. Opin. Nephrol. Hypertens..

[12] Grossmann C., Gekle M. (2009). New aspects of rapid aldosterone signaling. Mol. Cell. Endocrinol..

[13] Thomas W., Harvey B.J. (2011). Mechanisms underlying rapid aldosterone effects in the kidney. Annu Rev. Physiol..

[14] Gekle M., Freudinger R., Mildenberger S., Schenk K., Marschitz I., Schramek H. (2001). Rapid activation of Na^+^/H^+^-exchange in MDCK cells by aldosterone involves MAP-kinase ERK1/2. Pflug. Arch..

[15] McEneaney V., Dooley R., Yusef Y.R., Keating N., Quinn U., Harvey B.J., Thomas W. (2010). Protein kinase D1 modulates aldosterone-induced ENaC activity in a renal cortical collecting duct cell line. Mol. Cell. Endocrinol..

[16] Harvey B.J., Higgins M. (2000). Nongenomic effects of aldosterone on Ca^2+^ in M-1 cortical collecting duct cells. Kidney Int..

[17] Feldman R.D., Gros R. (2013). Vascular effects of aldosterone: Sorting out the receptors and the ligands. Clin. Exp. Pharmacol. Physiol..

[18] Gros R., Ding Q., Davis M., Shaikh R., Liu B., Chorazyczewski J., Pickering J.G., Feldman R.D. (2011). Delineating the receptor mechanisms underlying the rapid vascular contractile effects of aldosterone and estradiol. Can. J. Physiol. Pharmacol..

[19] Mihailidou A.S., Funder J.W. (2005). Nongenomic effects of mineralocorticoid receptor activation in the cardiovascular system. Steroids.

[20] Price D.A., Porter L.E., Gordon M., Fisher N.D., De’Oliveira J.M., Laffel L.M., Passan D.R., Williams G.H., Hollenberg N.K. (1999). The paradox of the low-renin state in diabetic nephropathy. J. Am. Soc. Nephrol..

[21] Zhang B., Liu Z., Chang Y., Lv R., Guo H., Qiang P., Shimosawa T., Xu Q., Yang F. (2025). Aldosterone-Induced Transformation of Vascular Smooth Muscle Cells into Macrophage-like Cells Participates in Inflammatory Vascular Lesions. Int. J. Mol. Sci..

[22] Nagata M. (2016). Podocyte injury and its consequences. Kidney Int..

[23] Mathew J.T., Patni H., Chaudhary A.N., Liang W., Gupta A., Chander P.N., Ding G., Singhal P.C. (2008). Aldosterone induces mesangial cell apoptosis both in vivo and in vitro. Am. J. Physiol. Ren. Physiol..

[24] Terada Y., Kobayashi T., Kuwana H., Tanaka H., Inoshita S., Kuwahara M., Sasaki S. (2005). Aldosterone stimulates proliferation of mesangial cells by activating mitogen-activated protein kinase 1/2, cyclin D1, and cyclin A. J. Am. Soc. Nephrol..

[25] Liao C.-W., Chou C.-H., Wu X.-M., Chen Z.-W., Chen Y.-H., Chang Y.-Y., Wu V.-C., Rose-John S., Hung C.-S., Lin Y.-H. (2020). Interleukin-6 plays a critical role in aldosterone-induced macrophage recruitment and infiltration in the myocardium. Biochim. Biophys. Acta (BBA)—Mol. Basis Dis..

[26] Huang L.L., Nikolic-Paterson D.J., Ma F.Y., Tesch G.H. (2012). Aldosterone induces kidney fibroblast proliferation via activation of growth factor receptors and PI3K/MAPK signalling. Nephron Exp. Nephrol..

[27] Bai M., Chen Y., Zhao M., Zhang Y., He J.C., Huang S., Jia Z., Zhang A. (2017). NLRP3 inflammasome activation contributes to aldosterone-induced podocyte injury. Am. J. Physiol. Ren. Physiol..

[28] Ding W., Guo H., Xu C., Wang B., Zhang M., Ding F. (2016). Mitochondrial reactive oxygen species-mediated NLRP3 inflammasome activation contributes to aldosterone-induced renal tubular cells injury. Oncotarget.

[29] Yuan Y., Huang S., Wang W., Wang Y., Zhang P., Zhu C., Ding G., Liu B., Yang T., Zhang A. (2012). Activation of peroxisome proliferator-activated receptor-γ coactivator 1α ameliorates mitochondrial dysfunction and protects podocytes from aldosterone-induced injury. Kidney Int..

[30] Meng X.M., Tang P.M., Li J., Lan H.Y. (2015). TGF-beta/Smad signaling in renal fibrosis. Front. Physiol..

[31] Ke Q., Shi C., Lv Y., Wang L., Luo J., Jiang L., Yang J., Zhou Y. (2022). SGLT2 inhibitor counteracts NLRP3 inflammasome via tubular metabolite itaconate in fibrosis kidney. FASEB J..

[32] Mulrow P.J. (1999). Angiotensin II and aldosterone regulation. Regul. Pept..

[33] Natale P., Palmer S.C., Navaneethan S.D., Craig J.C., Strippoli G.F. (2024). Angiotensin-converting-enzyme inhibitors and angiotensin receptor blockers for preventing the progression of diabetic kidney disease. Cochrane Database Syst. Rev..

[34] Agarwal R., Joseph A., Anker S.D., Filippatos G., Rossing P., Ruilope L.M., Pitt B., Kolkhof P., Scott C., Lawatscheck R. (2022). Hyperkalemia Risk with Finerenone: Results from the FIDELIO-DKD Trial. J. Am. Soc. Nephrol..

[35] Bakris G.L., Agarwal R., Anker S.D., Pitt B., Ruilope L.M., Rossing P., Kolkhof P., Nowack C., Schloemer P., Joseph A. (2020). Effect of Finerenone on Chronic Kidney Disease Outcomes in Type 2 Diabetes. N. Engl. J. Med..

[36] Sato A., Saruta T. (2003). Aldosterone breakthrough during angiotensin-converting enzyme inhibitor therapy. Am. J. Hypertens..

[37] Struthers A.D. (1996). Aldosterone escape during angiotensin-converting enzyme inhibitor therapy in chronic heart failure. J. Card. Fail..

[38] Amar L., Azizi M., Menard J., Peyrard S., Watson C., Plouin P.F. (2010). Aldosterone synthase inhibition with LCI699: A proof-of-concept study in patients with primary aldosteronism. Hypertension.

[39] Dwyer J.P., Maklad N., Vedin O., Monyak J., Myte R., Chertow G.M., Heerspink H.J., Little D.J. (2026). Efficacy and Safety of Baxdrostat in Participants with CKD and Uncontrolled Hypertension: A Randomized, Double-Blind, Placebo-Controlled Trial. J. Am. Soc. Nephrol..

[40] (2026). A Phase III, Randomised, Double-Blind Study to Assess the Efficacy, Safety and Tolerability of Baxdrostat in Combination with Dapagliflozin Compared with Dapagliflozin Alone on Chronic Kidney Disease (CKD) Progression in Participants with CKD and High Blood Pressure [Internet]. https://clinicaltrials.gov/study/NCT06268873.

[41] (2026). A Phase IIb, Randomised, Multicentre, Double-Blind Study to Evaluate the Effect of Baxdrostat in Combination with Dapagliflozin Compared with Baxdrostat on Albuminuria in Participants with Chronic Kidney Disease and High Blood Pressure [Internet]. https://clinicaltrials.gov/study/NCT07222917.

[42] (2024). A Phase III, Randomised, Double-Blind, Placebo-Controlled, Event-Driven Study to Assess the Efficacy, Safety and Tolerability of Baxdrostat in Combination with Dapagliflozin Compared with Dapagliflozin Alone on Renal Outcomes and Cardiovascular Mortality in Participants with Chronic Kidney Disease and High Blood Pressure [Internet]. https://clinicaltrials.gov/study/NCT06742723.

[43] (2026). A Randomized, Crossover, Double Blind, Placebo Controlled, Phase 2 Study to Evaluate the Efficacy and Safety of Lorundrostat in Addition to Sodium-Glucose Cotransporter-2 Inhibitors, in Adults With Hypertension and Chronic Kidney Disease with Albuminuria [Internet]. https://clinicaltrials.gov/study/NCT06150924.

[44] Tuttle K.R., Hauske S.J., Canziani M.E., Caramori M.L., Cherney D., Cronin L., Heerspink H.J.L., Hugo C., Nangaku M., Rotter R.C. (2024). Efficacy and safety of aldosterone synthase inhibition with and without empagliflozin for chronic kidney disease: A randomised, controlled, phase 2 trial. Lancet.

[45] (2026). A Phase II Randomised, Double-Blind, Parallel-Group, Multicentre, International Trial to Investigate the Safety and Efficacy of Vicadrostat and Empagliflozin Administered with Simultaneous vs Staggered Initiation in Participants with Chronic Kidney Disease at Risk of Kidney Disease Progression [Internet]. https://clinicaltrials.gov/study/NCT06926660.

[46] (2026). A Multicenter, International, Randomized, Double-Blind, Placebo-Controlled Clinical Trial of the Aldosterone Synthase Inhibitor BI 690517 in Combination with Empagliflozin in Patients with Chronic Kidney Disease [Internet]. https://clinicaltrials.gov/study/NCT06531824.

[47] Amornritvanich P., Anothaisintawee T., Attia J., McKay G.J., Thakkinstian A. (2025). Efficacy of Mineralocorticoid Receptor Antagonists on Kidney and Cardiovascular Outcomes in Patients With Chronic Kidney Disease: An Umbrella Review. Kidney Med..

[48] Queiroga F., Araújo B., Rivera A., Consoli L., Ujjawal A., Mansouri E.S., Akabane M.A.C.C., Barrera N.I., Ramos A.B.V., Iqbal A. (2026). Second-Generation Aldosterone Synthase Inhibitors for Hypertension: A Bayesian Meta-Analysis of Randomized Trials. JACC Adv..

